# Peroxide of Hydrogen as an Aid to Diagnosis

**Published:** 1893-04

**Authors:** 


### Peroxide of Hydrogen as an Aid to Diagnosis.

E. Stuver has suggested the injection of peroxide of hydrogen as an aid to diagnosis, in cases in which pus in a closed cavity is suspected. The injection of peroxide of hydrogen (Marchand's), with a small syringe, is very quickly followed by distention of the part when pus is present.

